# Peculiar Symptoms

**Published:** 1888-11

**Authors:** W. S. Gee

**Affiliations:** Chicago


### PECULIAR SYMPTOMS.

W. S. Gee, M. D., Chicago.

A practical question often asked by the embryonic homœopath, whether old or young, is this one: "How are we to know which are the leading symptoms upon which to base the prescription?" The character of the question demands a practical answer. In the answer we must suppose that the physician has fulfilled all previous requirements, such as thoroughly acquainting himself with the anatomy of the human body, the physiology; for how could he know the abnormal until he has learned to recognize at once the normal, and other collateral branches for similar reasons? He must know as well what is normal in the given case, else how should he know what is abnormal. He must have made a careful, minute history of the individual peculiarities in health, the heredity, early abnormal manifestations, life, business habits, likes and dislikes—in a word, a complete history of his unnatural symptoms. This history must include a detailed account of all symptoms of marked character which occurred in past illness as well as those of the present. The greatest and most difficult task has now been accomplished, as has been correctly stated by Hahnemann. We may well quote from this wise man in the 153d section of the *Organon:* "This search for the homœopathic, specific remedy, consists in the *comparison* of the totality of the symptoms of the natural disease with the lists of symptoms of our tested drugs, among which a morbific potency is to be found, corresponding in similitude with the diseases to be cured. In making this comparison, the more *prominent,* uncommon, and peculiar (characteristic) features of the case are especially, and almost exclusively, considered and noted; for *these, in particular, should bear the closest similitude to the symptoms of the desired medicine,* if that is to accomplish the cure. The more general and indefinite symptoms, such as want of appetite, headache, weakness, restless sleep, distress, etc., unless more clearly defined, deserve but little notice on account of their vagueness, and also because generalities of this kind are common to every disease and to almost every drug." Is this not a very definite and explicit answer to our question? Nothing short of the totality will suffice, but the method here indicated shortens the process of finding the desired remedy. Now our question resolves itself into this one: what *are* the "more *prominent,* uncommon, and peculiar features of the case?" Every person has the common marks of distinction by which he is classed as belonging to the human family. The millions of people on the earth in the normal state all have the upright form, have heads, arms, hands, legs, feet, eyes, mouths, tongues, etc., *and yet no two are alike in any one particular part.*

The great similarity enables them to be classified, and grouped as having common features, and yet one class differs from another, and is seen in the different races of men. Even in the same race there exists a marked difference in all the parts named, between those of the male and female of that race. What is stranger still, no two members of the same family are alike. No two brothers or sisters, born of the same parents, in the same climate, in the same house, even twins born at the same time and nursed by the mother, who partakes of her food from which the same milk is given to each, are ever found to be alike. In fact, many times the greatest dissimilarity may be found in instances of the latter. Neither are the two hands, feet, eyes, toes, nor fingers alike in the same individual.

Certainly great similarities exist, and from this fact have grown all organizations having a common interest, such as colonies, families, cities, states, governments, schools, churches, and associations of physicians—homœopathic in particular.

Our food is similar, so with our clothing, amusements, modes of exercise, business, etc., and yet there exist differences in every individual trait, so that these millions of people are *millions of individuals* with distinctive peculiarities. These peculiar marks may not remain the same at all times even in the same person. The same analogy exists in the lower animals and in the vegetable kingdom. The differences are always noted when we *individualize* them.

Thus far we have noted the peculiarities which exist in the *normal* condition. Does not the individual still have his peculiarities when he is sick? Surely, his normal distinguishing points may change with him and become abnormal symptoms. Disease is disturbed—disarranged life force the consequent result. As a train on a switch runs off the track and must be "backed" on, so with the misguided vital force. Our bodies are under the control of this vital dynamis, and they are composed of organs which may become diseased singly, collectively, and consecutively. The diseases are classified because of similarities either as to location, causes probable, or development. While many diseases are alike in some respects, as, *e. g.*, fevers, yet, in fact, no two are exactly alike. But very slight differences many times serve to distinguish one from another. The same disease varies in different individuals and even in the same person at different times. This is true of most diseases. The name of the disease is only for the convenience of the physician and friends. It conveys no definite idea as to what will prove curative in any given case. If the physician knows what is normal in general and in this individual in particular, he is now able to make the "comparison" according to the section quoted above. Diseases have special symptoms so grouped as to convey to the physician the category for the given case. The pathognomonic and concomitant symptoms suggest to him the name. If he is a true homœopathic physician he has all the facts necessary to make the so-called diagnosis, and for his protection and the satisfaction of the friends of the patient he may give the name.

This inspires the confidence of the family and enables him to indicate to them the probable prognosis. When we compare this record with the symptoms of the arbitrary disease we see at once the extra "uncommon, peculiar" ones as well as I note the common symptoms. We found in making the anamnesis that these "peculiar symptoms" were "prominent," unvarying, and had existed with the other unnatural symptoms. They must not be omitted from the totality of the disease symptoms. Hence, they must be included in the curative sphere of the appropriate remedy. We know that whatever remedy be chosen, it must include these symptoms, and thus we may shorten the process by looking at once to the one indicated by them, using the common ones later.

Suppose, for example, we take a case of intermittent fever. This disease is not uncommon. Our patient has had a chill, fever, and sweat, and applies for relief. We must learn more. Suppose he had no thirst during the chill—that is not strange or uncommon—but suppose he had *great thirst* while cold. That is uncommon and to some degree "peculiar;" make a note of that. He had thirst during the fever; that is common, but "absence of thirst" during the hot stage is "uncommon." He wanted to be covered during the chill—common; uncovered during the chill—uncommon, while the reverse is true in the fever. Suppose he sweats profusely after the fever, this is common, but absence of sweat uncommon. The absence of one stage is uncommon, so with a reverse order of them. The presence of a rash is a "peculiar" symptom with intermittent fever. If the patient has great thirst in all stages or the absence of it in all stages, in either case the symptom is unusual. If the patient sleeps during all stages it is worthy of special notice. So with all the symptoms recorded, the prominent, peculiar, uncommon ones requiring first consideration, but the common ones are necessary to make the totality.

We will now take as another example a case of scarlet fever. The ordinary cases of this disease include high fever, headache, sore, red, inflamed throat, with difficulty of swallowing, dry skin, and rash. Other symptoms might be included, but these are present in all marked cases of the disease. We cannot prescribe on these symptoms but must add to them some of an individual character. The presence of a rash is "peculiar" and "uncommon" in intermittent fever, but not so with scarlet fever, for here the absence of it would be "uncommon." The common symptoms of the disease are common to many remedies; but suppose another be added, such as "*picks the lips until they bleed*," and on inquiry we should find that to be a "prominent" symptom. Before the last symptom we could not decide, but the selection is now made with ease because Arum tri. includes the last "peculiar" *prominent* symptoms as well as the common ones. Why is the symptom "peculiar"? Because all cases of the disease do not have it, but only "peculiar" cases, and the remedy which meets the totality must include that symptom. Suppose another patient, having the same disease with the common symptoms has this extra or "uncommon" one—*The patient is terrified even to spasms when opening his eyes.* This is a very unusual symptom and one of a character not to be overlooked from the disease record. The remedy for this case is Stram., and no other will cure if the case is otherwise a fatal one—that is, one which will prove fatal if the indicated remedy is not given. The Stram. is selected for the totality, but the last symptom decides the choice. The "uncommon" symptoms may be *very* "peculiar" ones sometimes, and when taken alone are even ridiculous. A little patient twelve years old was taken very ill with a nervous difficulty, and, although naturally a very independent, dignified little girl, a strange freak presented by which the mother was obliged to take her on lap and sing to her to put her to sleep. She could not go to sleep otherwise. The case was carefully studied, and, the "relief from music" being such a prominent symptom, reference was made to Tarent. hisp. and it covered the totality and cured the case. A boy with a loose, watery diarrhœa had been troubled with it for a week and nothing he had taken had seemed to relieve him. He mentioned incidentally that he turned sick and was even obliged to leave the table "*if he saw or heard the water running from the hydrant.*" Hydrophob. cured him in twenty-four hours.

A lady called some time ago, and as she was soon to take a trip, she said, "I am always constipated when traveling. I wonder if you can give me anything to relieve me?" Platina covered the symptoms in this case.

Let it be borne in mind, further, that the above section states "peculiar (characteristic) *features*" of the given case. *Symptoms* and *features* may differ in this wise: Common symptoms may be so arranged as to become peculiar "features," and yet the symptoms be still common. For instance, "frontal headache, nausea, vomiting, and high fever" make peculiar "features" in a case, yet each as a symptom is not uncommon. The grouping of features make the peculiar part of the case which would direct us to the curative remedy in Verat. viride.

It frequently happens that the anamnesis or history will give the peculiar symptoms to have existed sometime in the past. Dunham reports a case in which he cured with Aloes—a headache in winter—given because the remedy was indicated for his diarrhœa in summer. Another in which he cured a case of deafness from milkcrust with Mez., because it was indicated for the previous milkcrust. These facts are confirmed in our every-day work. The same rule applies to all diseases and conditions; hence, how unreasonable to prescribe for a disease. Does it not appear too puerile to prescribe for a supposed pathological condition? Who can give the pathology in a case that causes one to be restless, while another cannot bear moving, when both have the same disease? Why are many better and others worse after sleep? Why are not all rheumatic patients affected by the weather? It has been said by some that such prescribers are "symptomatologists," who are not able to diagnose their cases, and that they prescribe on one or a few silly symptoms.

Of one of these might be asked: What are you but a "symptomatologist," and do you not prescribe on a "few symptoms"?

This difference may be noted: The former, if true to his work, takes *all* the symptoms, and he only may make the diagnosis from the facts at hand, but as he deems the selection of the remedy of more importance, his search is immediately begun, and when such remedy is found he is satisfied, rather than to spend time in theorizing and using misleading arbitrary names for the amusement of friends and for his personal aggrandizement. His objector needs but a few symptoms, looks into all the openings of his patient, stretches and compares them, thumps him for a time, gives some long name to the trouble, sends the patient to a drug store or "pharmacy" with a prescription calling for one drug or a mixture of drugs, which only palliate or suppress the symptoms giving most distress. To him, "uncommon," "peculiar" symptoms are but "chaff" and are "reflex." The results may be seen when chronic cases which do not "get well of themselves" fall under the care of such. Their failures cause some very "peculiar symptoms," such as seeking to "unite the two schools." They, never having received the light of Homœopathy, go groping about in darkness, and occasionally we hear a groan of "Hahnemanniacs," or "cranks," "bottle-washers." Not infrequently we learn that Hahnemann was in his dotage, was "visionary," and did not know much "physiology and pathology;" that his reported cures of cancers and other malignant troubles were fabulous, and that he could not detect a malignant condition. They have not read Hahnemann's books, yet they pass for "homœopaths."

After exhausting the latest allopathic measures, adopting germicides, and becoming liberal enough to swallow allopathy by degrees, such as Quinine for malaria, Mercury and Potassium iodide for syphilis, and other *drugs for diseases,* is it any wonder that *they* have found it necessary to "expunge the chaff" and "revise the *materia medica*"?

We speak of the "medical fraternity." Is it to-day a brotherhood in the highest sense? Is it not disappointing that we at this late day should see such internal dissensions within the "homœopathic" ranks? Has our cherished Hahnemann and the law of nature promulgated by him been honorably superseded? Is it not time to call a halt and see whether we are proving true to our trust? Can we not unite ourselves socially, or at least professionally, and work together to advance the true cause of the God-given boon, Homœopathy? As hinted in the body of this paper, can we detect the abnormal before we know the normal? Then is it reasonable for us to criticise or reject any of Hahnemann's instructions until we have thoroughly mastered the philosophy of Homœopathy as taught by Hahnemann? Let us study and become thoroughly familiar with his writings. Let us become as familiar with the *materia medica* as was Bœnninghausen, so cordially indorsed by Hahnemann, before we agree to reject a part of it. If we fall below the success of Hahnemann, Bœnninghausen, Hering, and others, it may be our fault. At least let us handle carefully that which has come down to us from the masters. Let us be slow to mar that which has cured so many thousands of sufferers before we were born.

A law of nature always acts *under certain conditions*. So with our law—the conditions must be understood. At any rate, let us "*be sure we are right, then go ahead.*"

---

DISCUSSION.

Dr. Hitchcock—When I first commenced the study of Homœopathy I commenced with the *Organon*, and I studied that for years before I attempted to make a prescription. There was no short or easy way of doing it. In the start I commenced by taking a complete history of my case as far as I was able, as I recognized that that was the only and the best way of doing it. I knew absolutely nothing of the *Materia Medica*, but by the diligent use of my repertory and study of the *Organon*, I began to prescribe, and from that day to this my prescriptions have all been made in exactly the same way, except in very acute cases where we can see at once what to do. I think that a great error in our method of prescribing is to memorize the remedies. I have made it a point never to memorize any remedy, and I do not believe I could give you the characteristics of a dozen remedies. Therefore I am not prejudiced, in the first coming of my case, and I attribute my success—what success I have had—to the fact that I am not prejudiced, and I think the young men, like myself, who have not had the years of experience, will find the best way is not to attempt to memorize but to absorb, and that will give us good homœopathic prescribers.

Dr. Holmes—If I call a man in consultation with me I want him to be able to see what is to be done. I do not want him to have to study a week on the case—that is not what I call him in for—but I want him to be able to tell me the remedy without resorting to his books. Now I would like to ask this question, Do you think a book prescriber is a desirable thing to be?

Dr. Hitchcock—I certainly do.

Dr. Wells.—I wish to give the opinion of a man who was an inmate of Hahnemann's family ten years and was his assistant, and the best assistant who ever walked the American soil. I suppose he has said to me a hundred times, "You do not know what you have to give your case until you study it," and that is true.

Dr. Kent—I always carry my books, and I generally have a satchel full of them. I do not mean most always, I mean *always.*

Dr. Allen—I want to tell Dr. Holmes what Dr. Guernsey once told me. A patient was seriously ill, and he wished to have Dr. Dunham in consultation. He lived in Philadelphia. In the evening they had a hasty lunch, and then went up to a room and sat down and carefully wrote down the symptoms of that case. Now, this is the way Dunham and Guernsey used to do.

Dr. Ballard—If Dr. Holmes should apply to a lawyer for advice, where a few dollars depended, he would not receive that opinion until that lawyer had first consulted his library. Now in regard to that book business, if Dr. Holmes does not think much of it, he would never call me in consultation a second time, for he will certainly find my repertory with me every time. There was a young man in Chicago who was very sick with double pneumonia. He was given up to die by his allopathic doctor. They telegraphed to his father, in New York, Dr. Finch. Dr. Finch came on, first getting my name. When he arrived he found his boy scarcely breathing. He came to see me and said, "I wish you would go over there with me." I went and sat down at the bedside with the old gentleman, and he says to me, "Don't hurry; this is my only boy; take your time." There was the boy almost breathing his last. I sat down at the bedside and took the case as carefully as I could on paper, and tried to think, and the Doctor said, "Don't worry yourself. I know where you stand. Go home and take your time with your books, and I will come over in the evening." He did come, and we two studied that case for two hours. It seemed that every minute that boy would breathe his last, but in spite of that we studied the case with the result that that boy is alive to-day and traveling about all right.

Dr. Wesselhœft—I suppose that Dr. Holmes is in the same position that we all have been in; that when he has a case that he thinks requires quick action, he wants a man who is ready for that quick action and save that patient's life, if possible, in as short time as possible. But those of us who have had any experience, and have gone through just such experiences as Dr. Holmes, I think must know that the majority of our dangerously sick cases in which we have just those feelings of being in a hurry to do something, have been lost on account of the hurry in which the prescription has been made.

Dr. Holmes—Now, you are all right; I don't doubt that in the least, and I am right, too, and I can prove it. Any man that will stand up here and tell me that it is no use to memorize the *materia medica*, I claim is wrong; it is wrong, and every man that does not do it, does wrong.

Dr. Wells—The gentleman thinks he is right, Mr. President; I think he is wrong. For many years I carried a satchel, and to this day I am not able to make a prescription until I have studied.

Dr. Biegler—I can say to Dr. Holmes that I can probably prescribe off-hand as well as any one in this room; but I have learned by experience the danger of doing that in the majority of cases. I carry not only a book, but carry a heavy satchel of books, so heavy that it requires my man to carry it for me, and I have never been degraded nor been looked upon with any disrespect for doing so. On the contrary, the good work that results from the use of those books is appreciated, even by those who are not considered the most intelligent.

Dr. Wells—It is a long time since I began to practice Homœopathy. Then we had proved drugs something less than two hundred. There was a man in London said to be able to recite the *materia medica* from beginning to end; he had memorized it; he was the poorest prescriber in all England.

Dr. Gee—The paper was intended to shorten the process of finding the remedy after taking the case. I may say that I carry a satchel of books when it is convenient to do so. I do not, as a rule, carry all of my books, because it is not always convenient. But in consultation work I use all of my books.—*Transactions I. H. A.*, 1888.

